# Effects of enhanced hydrological connectivity on Mediterranean salt marsh fish assemblages with emphasis on the endangered Spanish toothcarp (*Aphanius iberus*)

**DOI:** 10.7717/peerj.3009

**Published:** 2017-02-28

**Authors:** Patricia Prado, Carles Alcaraz, Lluis Jornet, Nuno Caiola, Carles Ibáñez

**Affiliations:** Aquatic Ecosystems, Institut de Recerca i Tecnologia Agroalimentàries, Sant Carles de la Ràpita, Tarragona, Spain

**Keywords:** Distance to the sea, Connectivity, Isolation, Salt marsh rehabilitation, Fish diversity, *Aphanius iberus*, Fish dispersal

## Abstract

The hydrological connectivity between the salt marsh and the sea was partially restored in a Mediterranean wetland containing isolated ponds resulting from former salt extraction and aquaculture activities. A preliminary assessment provided evidence that ponds farther from the sea hosted very large numbers of the endangered Spanish toothcarp, *Aphanius iberus*, suggesting that individuals had been trapped and consequently reach unnaturally high densities. In order to achieve both habitat rehabilitation and toothcarp conservation, efforts were made to create a gradient of hydrologically connected areas, including isolated fish reservoirs, semi-isolated, and connected salt marsh-sea areas that could allow migratory movements of fish and provide some protection for *A. iberus*. The fish community was monitored prior to, and for three years after rehabilitation. Results showed an increase in the number of fish species within semi-isolated areas (Zone A), whereas areas adjacent to the sea (Zone B) increased the number of marine species and decreased that of estuarine species (ES). Yet overall differences in fish assemblages were much higher between zones than among study years. Generalized linear models (GLMs) evidenced that distance to the sea was the most important variable explaining the local diversity of the fish community after restoration, with occasional influence of other factors such as temperature, and depth. The abundance of *A. iberus* was consistently higher in semi-isolated areas at greater distances from the sea, but a decline occurred in both zones and in isolated reservoir ponds after restoration efforts, which may be attributable to interannual differences in recruitment success and, to a lesser extent, to dispersal into adjacent habitats. A negative effect of restoration works on fish population cannot be excluded, but the final outcome of the intervention likely needs a longer period.

## Introduction

Estuarine and coastal ecosystems are among the most heavily exploited and threatened natural systems in the world (Worm et al., 2006; Halpern et al., 2008). The loss of coastal vegetation, biodiversity, and ecosystem functions has additionally favored biological invasions, decreased water quality, and increased erosion from flooding and storm events (Halpern et al., 2008; Koch et al., 2009). Such pervasive degradation of coastal marine ecosystems has led to considerable interest in their protection and rehabilitation (e.g., Matthews & Minello, 1994; Beck et al., 2003). In addition, protecting the habitat for endangered and socio-ecologically important species is also a central conservation strategy (see Ceballos, Rodríguez & Medellín, 1998; Noss, 2000).

Changes in the integrity of the landscape can modify hydrologic connectivity, disrupt key ecological functions and the life histories of a broad spectrum of organisms, and cause dramatic losses in aquatic biodiversity (Pringle, 2003). Among wetland ecosystems, salt marshes have been manipulated by humans since the Middle Ages through the construction of physical barriers that alter tidal action, as well as with agricultural practices and land uses that cause impermeability of top soil layers and alter natural biogeochemical functions (Portnoy & Giblin, 1997; Gedan, Silliman & Bertness, 2009). At the biotic level, such tidal restrictions and impoundments have been shown to reduce or eliminate habitat use by many invertebrate, fish, and bird species, particularly of those using marshes for spawning, nursery habitats, and for feeding migrations (Warren et al., 2002; Sheaves, 2009). Williams & Zedler (1999) found that fish assemblage composition was strongly associated to channel habitat characteristics, thus evidencing the importance of mimicking the natural hydrogeomorphology of the marsh when planning habitat rehabilitation projects. Enhanced tidal connectivity may also contribute to the effective control of alien plant species such as the common reed, *Phragmites australis*, which outcompetes native vegetation and may further reduce the abundance of fish juveniles and larvae (Able & Hagan, 2000). The size and structural connectivity between estuarine and marine ecosystems are also central variables explaining fish catch data for many groups of commercial species and make imperative the conservation spatial habitat features in order to maintain sustainable fish stocks (Meynecke et al., 2007).

The Ebro Delta (NW Mediterranean) constitutes an example of a highly modified human area, with ca. 65% of previous salt marsh–estuarine ecosystems now being devoted to rice cultivation (Benito, Trobajo & Ibáñez, 2014). Most of the remaining natural surface has been integrated into the Ebro Delta Natural Park, except for a fragment of salt marsh facing Alfacs Bay, which is not subjected to local management. This salt marsh habitat has been hydrologically isolated from the sea, as a result of former salt production and fish farming, and this may prevent migration and dispersal of aquatic fauna and alter the diversity and composition of local communities (Gedan, Silliman & Bertness, 2009).

The area is important because it hosts one of the main populations of the endangered Spanish toothcarp (*Aphanius iberus*), a cyprinodontid fish endemic to the Mediterranean coast of Spain that is considered in danger of extinction by the National Catalogue of Endangered Species and the Bern Convention on the Conservation of European Wildlife and Natural Habitats (Doadrio, 2002). *Aphanius iberus* is characterized by a high degree of isolation among its populations and is often abundant in salt pans (Oliva-Paterna, Torralva & Fernández-Delgado, 2006), such as in the salt marsh area described here, presumably because of natural dispersion from other bay regions and reduced competition with local and non-indigenous species at high salinities (Alcaraz & García-Berthou, 2007a; Alcaraz, Bisazza & García-Berthou, 2008). In addition, it typically displays a short life span (0–2 years; García-Berthou & Moreno-Amich, 1992) and important variability in interannual recruitment (Fernández-Delgado et al., 1988; Vargas & De Sostoa, 1996).

Enhancing the hydrologic connectivity of the study area was one of the main goals of a wider project supported by the Life-Nature Program of the European Union aimed at rehabilitating two coastal lagoons in the Ebro Delta to the condition existing before major human intervention. However, this goal was potentially in conflict with the interest of the Ebro Delta Natural Park in preserving the Spanish toothcarp population within isolated ponds. Restoration works were preceded by a preliminary assessment of the population, aimed at assessing whether the abundance of individuals was determined by environmental factors (e.g., salinity), historic reductions in the number of hydrologic connections, or a combination of both. Results (also included in this study) indicated that factors related to human-made isolation, such as distance to the sea and the presence or absence of an artificial concrete bottom, were the most relevant in determining toothcarp densities. Then, we tested whether rehabilitation and conservation needs could be reconciled by designing a gradient of hydrologically connected areas, including isolated, semi-isolated, and shoreface-connected salt marsh-sea that could allow fish migration and yet provide a variable degree of confinement for *A. iberus*. The total fish community, including *A. iberus*, and the local environmental variables were monitored before and during three consecutive years after restoration efforts in order to track possible changes in fish assemblages and to elucidate causes of variability. More specifically, we hypothesized that: (1) enhanced hydrological connections will increase the diversity and richness of fish species in the salt marsh area; (2) restoration will favor the development of distinctive assemblages in terms of fish abundance and composition; and (3) isolation (in terms of distance from the sea) will still be a central variable controlling the diversity and structure of fish assemblages after restoration, with some influence of other environmental variables. For *A. iberus*, a decrease in the overall abundance of individuals was also expected due to potential dispersal throughout the salt marsh and into Alfacs Bay, although with higher numbers at farther distances from the sea due to the species’ preference for isolated areas.

## Materials and Methods

### Study area and restoration works

**Figure 1:**
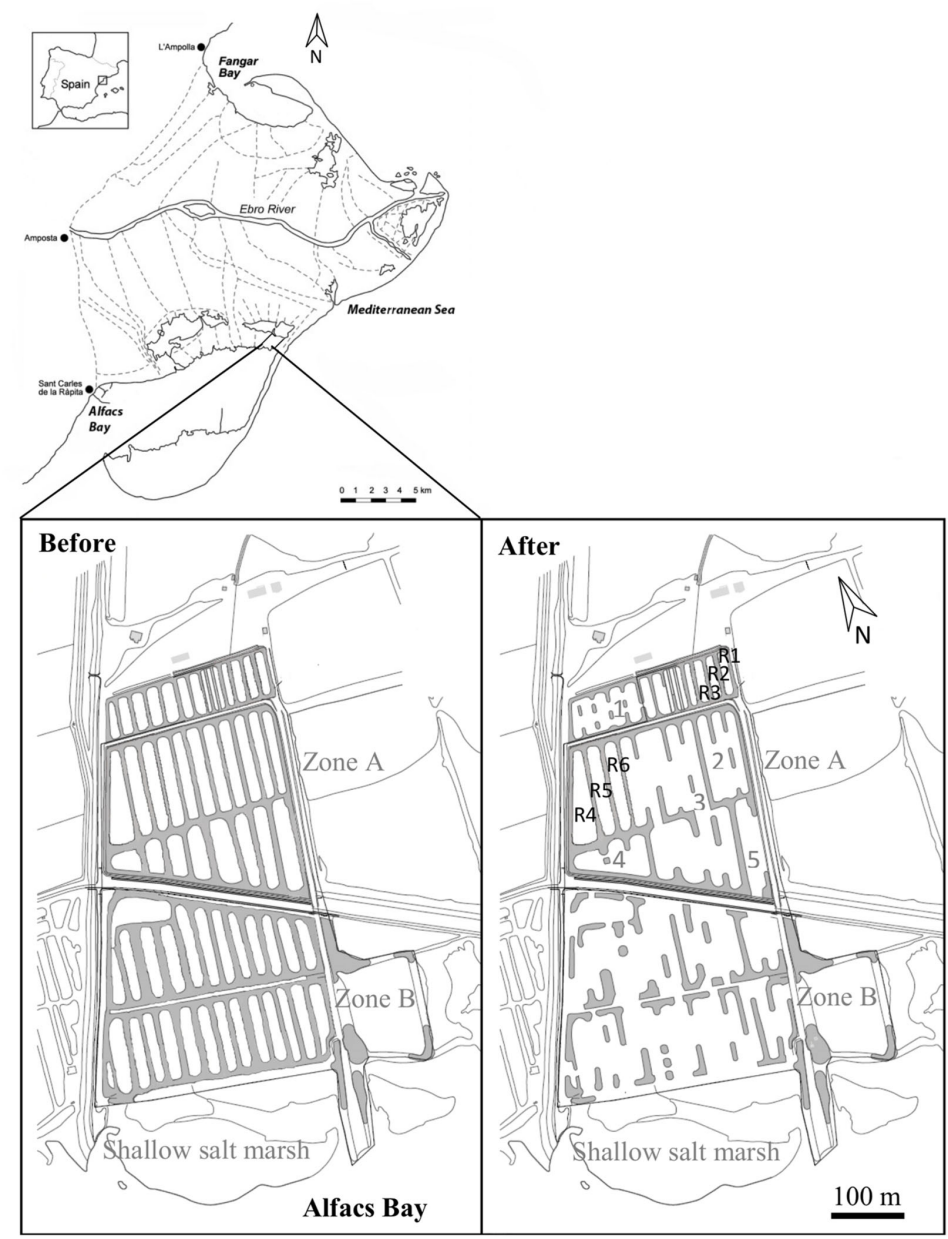
Location of the study area in the Ebro Delta, NW Mediterranean, and detail of the pond structure before and after restoration efforts in 2011. The two study zones (A and B) above and below the main traversing road are indicated. Remaining coalescent ponds in restored Zone A are numbered. Reservoir ponds are indicated from R1 to R6.

The Sant Antoni salt marsh area comprises 147 ha (ca. 1.5% of the Ebro Delta Natural Park) of *Salicornia* marshes and shallow ponds lying between the southern edge of the Tancada Lagoon and Alfacs Bay (Fig. 1). It is managed by private owners and constitutes one of the last fragments of impounded salt marsh habitat in the Ebro Delta. Before agricultural development in the 19th century, salt marshes stretched along the whole northern shore of the Alfacs Bay and connected it with the Tancada lagoon and another adjacent lagoon system (see Supplemental Information 3). After agricultural development, the remaining salt marsh was used for salt and aquaculture production, which deeply altered the natural regime of seawater flooding (Ibáñez et al., 1997). Prior to habitat rehabilitation in 2011–2012, the study area consisted of 60 aquaculture ponds, some isolated and some interconnected (Fig. 1). A road separates the area into two zones (A, B) that differ in distance from the sea and connectivity with it (Fig. 1).

A preliminary assessment was first conducted to determine whether the high abundance of *A. iberus* was due to natural factors or a consequence of human isolation in artificial ponds. This information was critical for deciding the most appropriate restoration strategy to recover connectivity functions while preserving the *A. iberus* population. After the assessment, land works were conducted during 4 months between November 2011 and February 2012, using shovel loaders and crawler excavators in order to enhance connectivity with the sea, particularly in Zone B. Six isolated ponds within Zone A were left intact as reservoirs for *A. iberus* and the rest were combined into five large ponds (Fig. 1). Extracted soil from the margins of aquaculture ponds was used to partially fill ponds, reducing water depth across the study area. The works were conducted from inland southwards toward the bay, and extreme care was taken during the reallocation of the extracted soil, in order to allow local fish and other benthic vagile fauna to move toward an adjacent area connected to another pond or bay canal. The fringing vegetation, before and after enhancement of hydrological connections, was a diverse community of halophytes and protected species of *Limonium* spp., whereas the submerged vegetation within ponds was mostly dominated by the spiral ditchgrass, *Ruppia cirrhosa*, and marine algae, particularly during the summer period. Removal of land around the ponds’ perimeter was also done taking into consideration, as much as possible, the distribution of *Limonium* spp. populations, which are protected by law.

### Fish sampling and environmental monitoring

A field permit for fish capture was granted by the Ministry of Agriculture, Livestock, Fisheries and Food (ref. SF/041), which also provided logistical support and supervision at each sampling event once per year during four consecutive years (2011–2014). No individuals were sacrificed for the purpose of this study, and all fish were released at the site of capture immediately after species identification. During the multiple years of the project (2011–2014), April was chosen for sampling in order to prevent disturbance of *A. iberus* individuals during the breeding season, which occurs mainly from May to September (Fernández-Delgado et al., 1988; Vargas & De Sostoa, 1996). Fyke nets and not ponds, which were only present in 2011, were used as replicate units throughout the study period. All fyke nets were 1.4 m long with a hoop diameter of 0.5 × 0.35 m and 4.0 mm mesh size, appropriate for all benthic species at shallow depths and those using the vegetation for sheltering during the day. Depth was very shallow across the entire study area and fyke nets traversed the whole water column avoiding otherwise potential bias in the capture of benthic vs. more pelagic species. Sampling took place only once per year in each zone. All fyke nets were set in late afternoon and hauled the next morning thanks to a team of local volunteers, hence having an average soaking time of 12 h. The next day, all nets were removed and all the fish captured were sorted to species level and counted. Individuals of the locally abundant green crab *Carcinus maenas* that were present within the fyke net were also counted. In 2011, three fyke nets per pond were deployed across the study area (*N* = 60 ponds, all of them sampled). Of the total of 180 nets, 95 in Zone A and 63 in Zone B were recovered and the rest were stolen by poachers. In the three following years, the numbers of replicate nets recovered were 81 and 50 in 2012, 75 and 43 in 2013, and 61 and 38 in 2014, in Zone A and Zone B respectively. Fyke nets were consistently placed more than 30 m apart in order to maximize the sampling of benthic surface accordingly to the potential home range of *A. iberus*. Although the home range of this species has not yet been studied, previous research suggests that it could be lower than 30 m, and similar distances are reported for other members of the Cyprinodontidae family such as *Fundulus heteroclitus* and *Fundulus luciae* (Able, Hagan & Brown, 2006; Alcaraz, Pou-Rovira & García-Berthou, 2008). In all, replicate numbers were much higher than required, since the low number of species in the area resulted in a stable yield/effort curve at only 10 fyke nets per zone each year.

Physicochemical variables of water (pH, temperature, salinity, and dissolved oxygen (ODO)) were measured within a 2-h range with a YSI 6660 multiparametric probe (equipped with a 650 MDS data logger) placed beside each fyke net. Dissolved nutrients (NOx, NH_4_, and PO_4_) were collected with 100 mL water bottles and kept frozen at −20 °C until determination following the Koroleff method (Koroleff, 1977). Depth was measured to the nearest mm using a meter stick at the center of each pond (2011) and beside the mouth of each fyke net (2012–2014). Distance to the sea was calculated with Google Earth as the minimum distance that a fish would have to travel from a given pond to reach the sea (2011), and from the georeferenced points where physicochemical variables were collected to the sea (2012–2014). Abundance of submerged and riparian vegetation was visually estimated as percent cover (0, 25, 50, 75, and 100) in sites where fyke nets were deployed. The type of substrate was assessed as presence/absence of natural vs. artificial substrate (i.e., concrete), and the slope of the ponds (2011) or basin area (2012–2014) was determined as the angle between the ground and the vertical. A full list of investigated environmental variables with mean values obtained per zone and year is provided in Table 1.

**Table 1:** Environmental variables (mean ± standard error) measured in Zone A and B of the study area before (2011) and after (2012–2013) habitat restoration. The number of connections and substrate type per pond is indicated prior pond removal in 2011. Nutrient data from 2014 are not available (NA).

Environmental variables	Zone A				Zone B			
	2011	2012	2013	2014	2011	2012	2013	2014
Distance to sea (m)	650.5 ± 21	698.8 ± 13.8	694 ± 22.1	757.7 ± 13.5	308.4 ± 16.5	245.8 ± 20.5	337.9 ± 23.4	356.8 ± 14.3
Pond connections (*N*)	0.9 ± 0.1				1.18 ± 0.1			
Natural/Artificial substrate	50/2				17/3			
Depth (cm)	41.5 ± 1.3	53.6 ± 1.6	55.0 ± 0.5	65 ± 1.6	44.5 ± 1	45.5 ± 2	46.6 ± 2.7	53.8 ± 1.7
Salinity	45.1 ± 2.8	45.2 ± 2.1	41.7 ± 2	39.8 ± 2.1	35.8 ± 1.2	34.8 ± 1.9	35.5 ± 0.1	36.1 ± 0.2
Water T °C	20.6 ± 0.08	14.9 ± 0.2	17.7 ± 0.5	22.6 ± 0.1	19.3 ± 0.1	16.8 ± 0.6	19.6 ± 0.1	25.6 ± 0.1
ODO (mg/L)	6.8 ± 0.4	9.5 ± 0.1	6.2 ± 0.2	9.4 ± 0.2	6.5 ± 0.3	9.9 ± 0.2	7.1 ± 0.07	11.6 ± 0.6
pH	8.1 ± 0.07	8.4 ± 0.04	8.1 ± 0.03	8.2 ± 0.02	8.2 ± 0.1	8.3 ± 0.03	8.1 ± 0.01	8.3 ± 0.1
NO_3_ (μg/L)	27.9 ± 6.5	30.8 ± 5.2	16.9 ± 6.2	NA	39.1 ± 10.5	33.1 ± 4.2	29.2 ± 6.8	NA
NO_2_ (μg/L)	5.5 ± 1.9	5.2 ± 1.2	7.1 ± 2.5	NA	5.7 ± 1.5	11.1 ± 2	9.6 ± 2.7	NA
NH_4_ (μg/L)	64.0 ± 11	86.6 ± 12	99.8 ± 26.1	NA	65.4 ± 14.5	90.7 ± 15.7	100.9 ± 26.1	NA
PO_4_ (μg/L)	19.4 ± 3.6	9.3 ± 3.6	17.7 ± 4.4	NA	13.2 ± 2.4	15.5 ± 1.3	8.8 ± 1.3	NA
Submerged veg. (%)	40.8 ± 7.2	28.0 ± 6.2	28.2 ± 4.8	62.7 ± 4.9	62.4 ± 4.1	10.6 ± 2.4	7.5 ± 1.7	52.2 ± 5.6
Riparian veg. (%)	80 ± 5.8	51.1 ± 6.5	31.6 ± 5.1	55.3 ± 4.6	90.9 ± 3.9	29.1 ± 5.8	8.75 ± 2.01	23.6 ± 4.2
Slope (%)	33.6 ± 2.1	34.5 ± 2.3	32.3 ± 1.5	35 ± 1	31.8 ± 2.3	31.0 ± 1.6	29.6 ± 2.5	25 ± 0

The percent abundance of fish species present at each study year was assigned to one of the following ecological guilds adapted from former classifications (França et al., 2012): (i) estuarine species (ES—highly euryhaline species resident within the estuary), (ii) marine species (MA—highly euryhaline species capable of moving from the sea throughout the full length of the estuary. Due to the low number of species present, this included marine seasonal migrants, marine juvenile migrants, and marine adventitious visitors), and (iii) catadromous species (C—freshwater species that use estuaries as migration pathways toward the sea).

Species richness was calculated as the total number of species observed per fyke net, and species diversity was estimated from the Shannon index (hereafter SI) such that:

$${\rm{H'}} = -\mathop \sum \limits_{i = 1}^s {p_i}\log {p_i}$$

where *s* is the total number of species and *p_i_* is the proportion of species *i* observed in the sample.

### Data analyses

#### Preliminary assessment

The association of toothcarp CPUE and fish community diversity (SI) with environmental (depth, distance to the sea, salinity, T °C, ODO, pH, slope and type of substrate) and biotic variables (riparian and submerged vegetation cover) before restoration efforts (2011) was analyzed with generalized linear models (GLMs), assuming a Gaussian error and the identity link function. Among biotic variables, accompanying species present at abundances higher than 1% of the total capture—*C. maenas*, *Potamochistus microps*, and *Atherina boyeri*—were also included. Zone was not used as a variable for the analyses because of its relationship with distance to the sea, which was used instead.

An information-theoretic approach was used to find the best approximating models (Burnham & Anderson, 2002). GLMs were built including all possible combinations of environmental and biotic variables, excluding interactions, due to the large number of variables included. Two additional criteria were used to define the candidate models: only those performing significantly better than the null model and those with a variance inflation factor of ≤5 were selected, in order to avoid multicollinearity effects in regression models (Ibáñez et al., 2012). The degree of support for each candidate model was assessed with the second order Akaike information criterion (AIC) (AICc), rescaled to obtain *Δ*AICc values (*Δ*AICc = AICc_i_ − minimum AICc). According to Burnham & Anderson (2002), models having *Δ*AICc values within 1–2 of the best model have the most substantial support, those within ca. 4–7 have considerably less support, while models with *Δ*AICc > 10 have either essentially no support and might be omitted from further consideration. Then, the relative plausibility of each candidate model was assessed by calculating Akaike’s weights (*w*_i_); *w*_i_ ranges from 0 to 1, and can be interpreted as the probability that a given model is the best model in the candidate set. Because no model was clearly the best one (i.e., *w*_i_ ≥ 0.9), we calculated model-average regression coefficients as the result of a weighted average (by model *w*_i_) of the regression coefficients across all models in which a given variable is present. No significant differences were observed between *Δ*AICc = 2 or *Δ*AICc = 7, and the later was used as supporting criteria. The relative importance of each independent variable was also calculated by the sum of *w*_i_ for all models in which a given variable occurs (Burnham & Anderson, 2002). Finally, model-averaged estimates were compared with regression coefficients from the full model to assess the impact of model selection bias on parameter estimates (Whittingham et al., 2005). For all of candidate models, residuals showed to be normally distributed according to the Shapiro–Francia normality test (*W* ≥ 0.97, *P* ≥ 0.33).

Prior to analysis, quantitative variables were transformed to improve linearity and homoscedasticity. Analyses were performed with R software version 3.1; the MuMIn 1.15.6 package was used for multi-model inference analysis.

#### Fish community

A two-way ANOVA was used to investigate differences between fish species richness and diversity (SI) among years and between zones (Year and Zone as fixed factors).

As in the preliminary assessment, an information-theoretic approach was used for assessing the relationship between the SI and environmental and biotic variables (except substrate type) at each study year after restoration, and for selecting the best approximating GLMs (Burnham & Anderson, 2002; Ibáñez et al., 2012). Since the time lag between sampling events (once a year) was sufficiently long for considering independence between them, we built separate models for each study year. Isolated reservoir ponds were excluded from all the 2012–2014 analyses since they were not subjected to restoration.

The importance of temporal and spatial changes in the structure of fish assemblages after restoration works was investigated for the whole temporal series (2011–2014) using the PRIMER v6 software package (Clarke & Gorley, 2006). Non-metric multidimensional scaling (nMDS) ordinations were used first used to obtain a visual representation of assemblages’ groupings among years and zones, and further ANOSIM analyses were conducted to quantify the importance of observed differences. All multivariate analyses also included the green crab (*C. maenas*) due to its high abundance and the potential disturbance of soft sediments and submerged vegetation it can cause (Ropes, 1968; Davis, Short & Burdick, 1998), which could negatively affect hatching of *A. iberus* on benthic macrophytes (Clavero, Blanco-Garrido & Prenda, 2007). All samples were standardized by the mean divided by standard deviation and log (*x* + 1) transformed prior to the analyses.

#### Spanish toothcarp population

Temporal and spatial trends in the abundance of *A. iberus* before and after restoration works were investigated with a two-way ANOVA, with Year (2011–2014) and Zone (A and B) as fixed factors. The effect of natural factors not related to salt marsh rehabilitation in the abundance of *A. iberus* within isolated reservoir ponds was investigated with a two-way ANOVA, with year (2011–2014) and pond (six levels) as fixed factors, due to specific location needs. Student–Newman–Keuls (SNK) post hoc tests were conducted for all significant ANOVA factors.

The association between *A. iberus* abundances and environmental and biotic variables at each study year after restoration was also investigated using an information-theoretic approach to find the best approximating models (Burnham & Anderson, 2002) following the same criteria described for the fish community and the preliminary assessment.

## Results

### Preliminary assessment

The AICc-based model selection suggested 322 plausible models (*Δ*AICc < 7) to explain variability in *A. iberus* abundance within ponds (Table 2A). The best model contained substrate type, distance to the sea, and *A. boyeri* plus the green crab *C. maenas* as accompanying species (see later for selection criterion). In contrast, the diversity of the local assemblages was shown to be mostly influenced by salinity, depth, and the percent cover of riparian vegetation, although patterns were less strong than those observed for *A. iberus* (Table 2B).

**Table 2:** Results from the information–theoretic framework analyses aimed to evaluate the importance of environmental and biotic variables in: (A) *A. iberus* abundance within ponds; and (B) the diversity of the fish community (SI) before reconstruction works in 2011. Model-averaged regression coefficients (*β*) are parameter coefficients averaged by model weight across all candidate models (*Δ*AICc < 7) in which the given parameter occurs; selection probability (SP) indicates the importance of an independent variable, and parameter bias is the difference between *β* and the full model coefficients. The number (*N*) of candidate models (*Δ*AIC < 7) and Pearson’s correlation coefficient (r) between observed and model predicted values is also shown.

A) *A. iberus* abundance	Averaged model *N* = 322, *r* = 0.78		
	*β*	SP	Bias
**Intercept**	**−6.018**		**0.126**
**AB**	**0.550**	**0.630**	**−0.098**
**CM**	**−1.401**	**1.000**	**0.122**
Water depth (cm)	−2.226	0.373	−0.058
Dissolved oxygen (mg/L)	0.238	0.176	1.258
**Distance to sea (m)**	**1.545**	**0.621**	**0.514**
PM	0.311	0.571	−0.103
Riparian vegetation (%)	−0.033	0.207	0.496
Salinity	0.744	0.233	−0.111
Submerged vegetation (%)	−0.056	0.451	0.162
**Substrate type**	**−1.037**	**0.974**	**0.127**
Water temperature (°C)	13.234	0.379	0.544

B) SI	Averaged model *N* = 79, *r* = 0.66		
	*β*	SP	Bias
Intercept	0.511		2.194
Distance to sea (m)	−0.076	0.280	−0.069
Dissolved oxygen (mg/L)	0.158	0.402	−0.018
**Water depth (cm)**	**0.445**	**0.770**	**−0.069**
**Riparian vegetation (%)**	**0.012**	**0.601**	**0.042**
**Salinity**	**−0.565**	**1.000**	**0.056**
Submerged vegetation (%)	0.002	0.203	1.181
Water temperature (°C)	−0.313	0.217	2.931
Substrate type	0.029	0.231	0.005

**Notes:**

Parameters included in the best model are indicated in bold.

AB, *A. boyeri*; CM, *C. maenas*; PM, *P. microps*.

### Fish community

**Figure 2:**
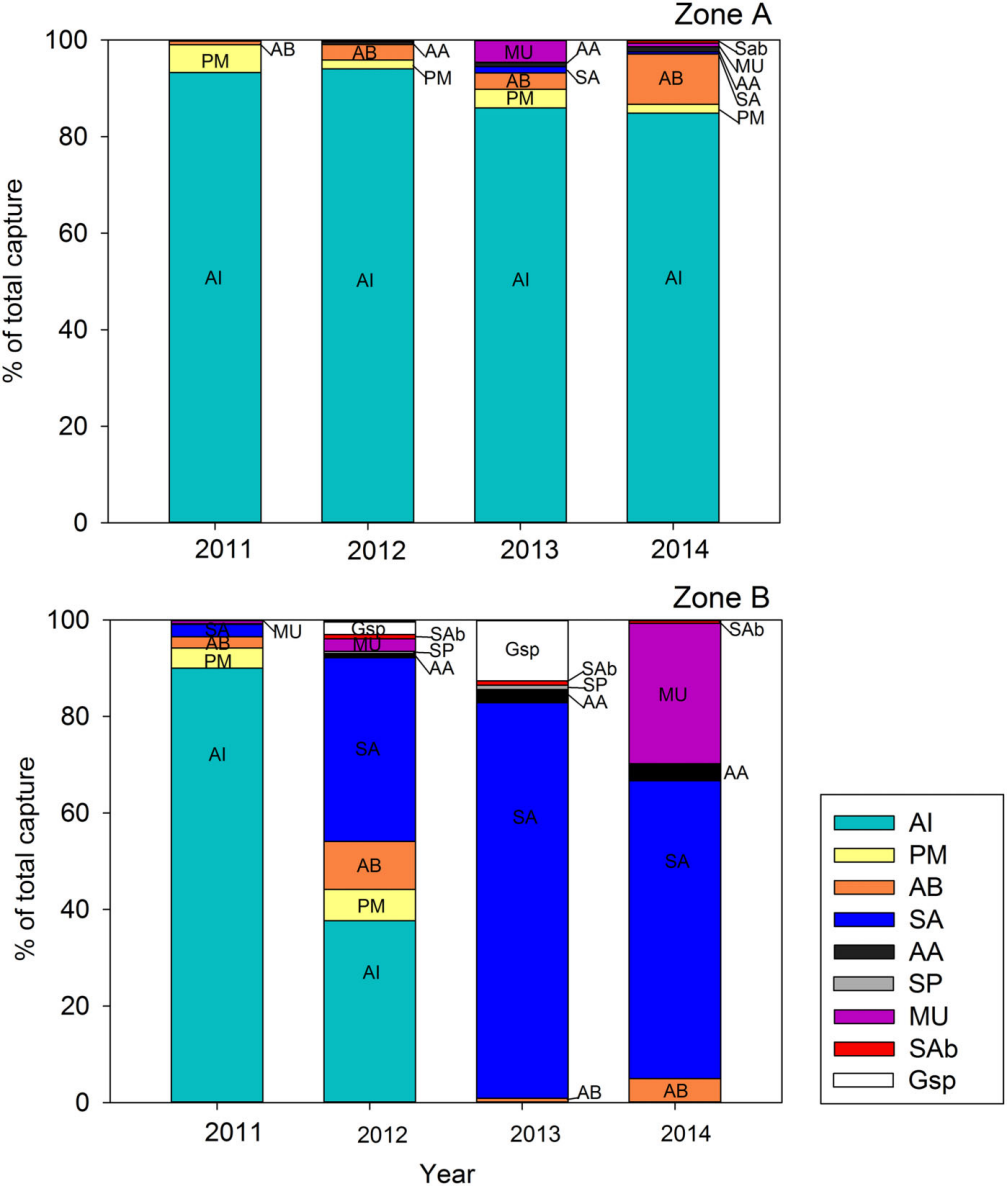
Percent abundance of fish species at each study year (2011–2014) and zone (A and B). AA, *A. anguilla* (C); PM, *P. microps* (ES); AB, *A. boyeri* (ES); AI, *A. iberus* (ES); SA, *S. aurata* (MA); MU, mullets (MA); SP, *S. pavo* (ES); SAb, *S. abaster* (ES); Gsp, *Gobius* sp (ES). Ecological guild categories (see text) are indicated in brackets. Other species observed in very low abundances and are not indicated. N_2011_ = 39,739 (A) and 3,100 (B) individuals; N_2012_ = 10,286 (A) and 231 (B) individuals; N_2013_ = 7,381 (A) and 111 (B) individuals; N_2014_ = 2,072 (A) and 141 (B) individuals.

A total of 14 fish species were found over the two salt marsh zones during the four study years (see abundances in Fig. 2). They belonged to nine different families: Anguillidae (*Anguilla anguilla*); Cyprinodontidae (*A. iberus*); Fundulidae (*Fundulus heteroclitus*, only one individual in 2013); Atherinidae (*A. boyeri*); Mugilidae (*Mugil cephalus*, and *Liza* sp.); Blenniidae (*Salaria pavo*); Gobiidae (*P. microps*, *Gobius geniporus*, and *Gobius niger*); Sparidae (*Sparus aurata*); Moronidae (*Dicentrarchus labrax*); and Sygnatidae (*Syngnathus abaster*, and *Syngnathus acus*). Additionally, invertebrate species such as the green crab (*C. maenas,* Portunidae) and shrimp (*Palaemonetes* sp.; Palaemonidae) were also captured in abundance.

Fish assemblages in Zone A were dominated by ES (mostly *A. iberus* and *P. microps* to a lesser extent; see codes in Fig. 2), with similar abundances from 2011 (ca. 99%) to 2014 (ca. 98%) and experienced a slight variations in the abundance of MA (ca. 0.1% in 2011, 0.25 in 2012, 6% in 2013, and 1.2% in 2014). In Zone B, the abundance of ES (*A. iberus* and *P. microps*) decreased sharply from throughout the study period (from ca. 96% in 2011 to 58% in 2012, 15% in 2013, and 6% in 2014). In contrast, the abundances of MA and to a lesser extent that of C species experienced the reverse pattern from ca. 3% in 2011 to 41% in 2012, 82% in 2013, and 91% in 2014).

**Figure 3:**
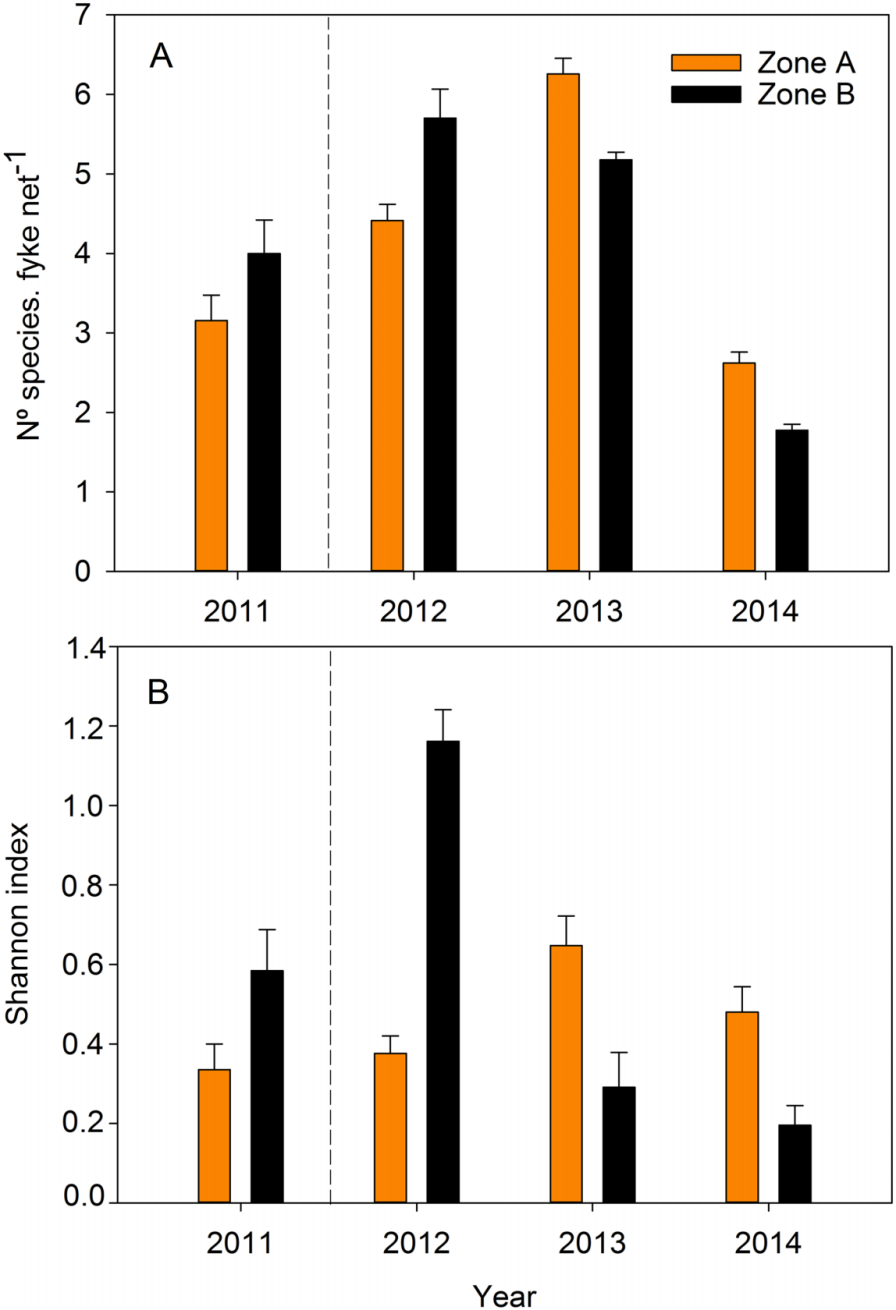
(A) Species richness, and (B) Shannon diversity index for fishes and crustaceans collected with fyke nets in the two zones of the study area during the four study years. The dotted line indicates the moment at which restoration took place between 2011 and 2012. Error bars are the standard error.

Species richness and the SI displayed significant differences among study years (Species richness: *F*_3, 439_ = 93.52, *P* < 0.001, in SNK: 2013 > 2012 > 2011 > 2014; SI: *F*_3, 439_ = 9.92, *P* < 0.001, in SNK: 2012 ≥ 2011 = 2013 = 2014) but not between zones (*P* = 0.79, and *P* = 0.07, respectively for species richness and SI). There was a significant Year × Zone interaction, with Zone A showing lower richness and SI in 2011–2012, and higher in 2013–2014 (Species richness: *F*_3, 439_ = 10.08, *P* < 0.001; SI: *F*_3, 439_ = 19.77, *P* < 0.001; see Figs. 3A and 3B).

Predictive models from the information–theoretic framework analysis (*N* = 2–67 with *Δ*AICc < 7) consistently selected distance to the sea (2012–2014) and temperature (except for 2013) as important variables explaining variability in the SI. Water depth was also a significant variable, but only in the 2012 model (Table 3).

**Table 3:** Results from the information-theoretic analyses showing the importance of environmental and biotic variables in the Shannon index (SI) after restoration works (2012–2014), see Table 2 for details.

Model Parameter	2012			2013			2014		
	*N* = 44, *r* = 0.78			*N* = 2, *r* = 0.36			*N* = 67, *r* = 0.39		
	*β*	SP	Bias	*β*	SP	Bias	*β*	SP	Bias
Intercept	0.866		1.742	−0.266		0.861	1.678		−0.308
**Dist. to sea (m)**	**−0.288**	**0.991**	**0.138**	**0.848**	**1.000**	**0.187**	**0.138**	**0.575**	**−0.126**
ODO (mg/L)	0.697	0.300	−0.283				0.106	0.291	−0.160
**Depth (cm)**	**−0.380**	**0.657**	**0.0096**				−0.173	0.365	−0.218
Riparian veg. (%)	0.001	0.163	−0.150				0.002	0.241	0.746
Salinity (PSU)	−0.149	0.203	1.763				0.334	0.266	1.334
Submerged veg. (%)	0.003	0.201	−0.357	0.315	0.371	−0.894	0.001	0.226	0.887
**Water T °C**	**0.632**	**0.461**	**−0.329**				**−1.457**	**0.901**	**−0.019**

**Note:**

Parameters included in the best model are highlighted in bold.

**Figure 4:**
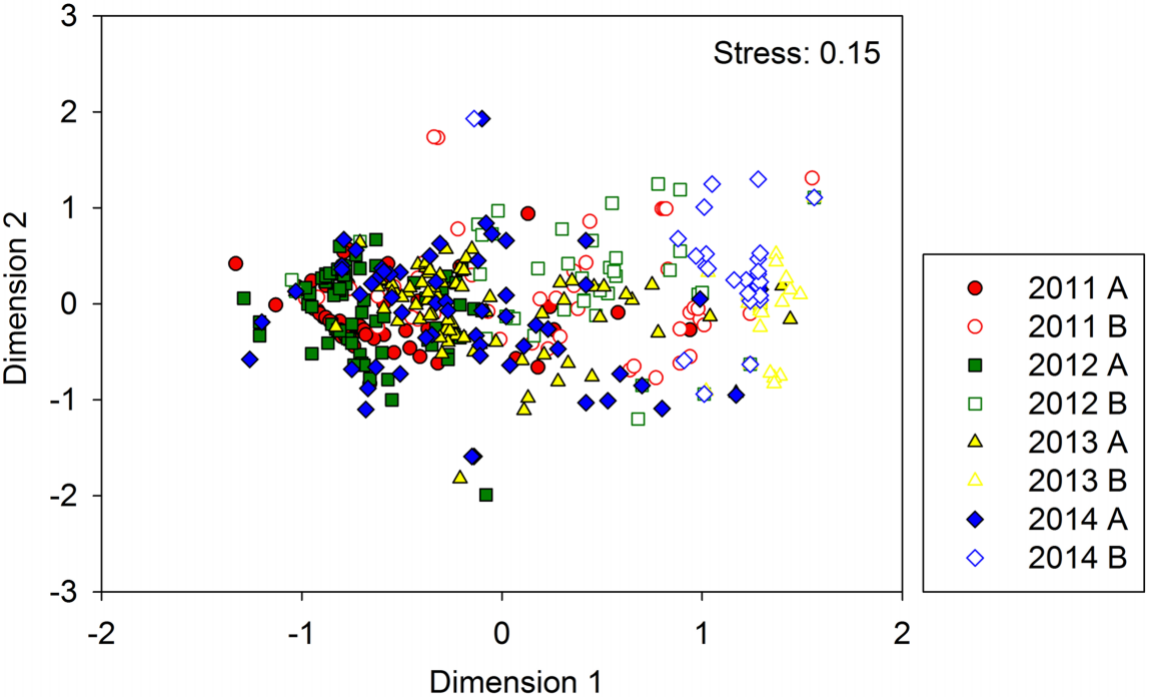
nMDS ordination showing differences in benthic assemblages (fish and crustaceans) found at the study area (Zones A and B) during the four year study period. Data were Log (x + 1) transformed.

nMDS ordination showed no apparent groupings for samples belonging to each study year, but there were some differences between Zones A and B (Fig. 4). Further, ANOSIM results confirmed the low importance of annual differences (*R* = 0.196, *P* = 0.001) and the stronger significance of spatial differences between zones (*R* = 0.541, *P* = 0.001). In pair-wise comparisons, differences between 2011 and 2012 were the lowest (*R* = 0.043, *P* = 0.001), and increased during the two following years (*R* = 0.33 and *R* = 0.30, *P* = 0.001, respectively for 2011–2013 and 2011–2014). Differences between 2012–2013 and 2012–2014 were small (*R* = 0.23 and *R* = 0.19, *P* = 0.001, respectively), and larger than those of 2013–2014 (*R* = 0.086, *P* = 0.001).

### Spanish toothcarp population

**Figure 5:**
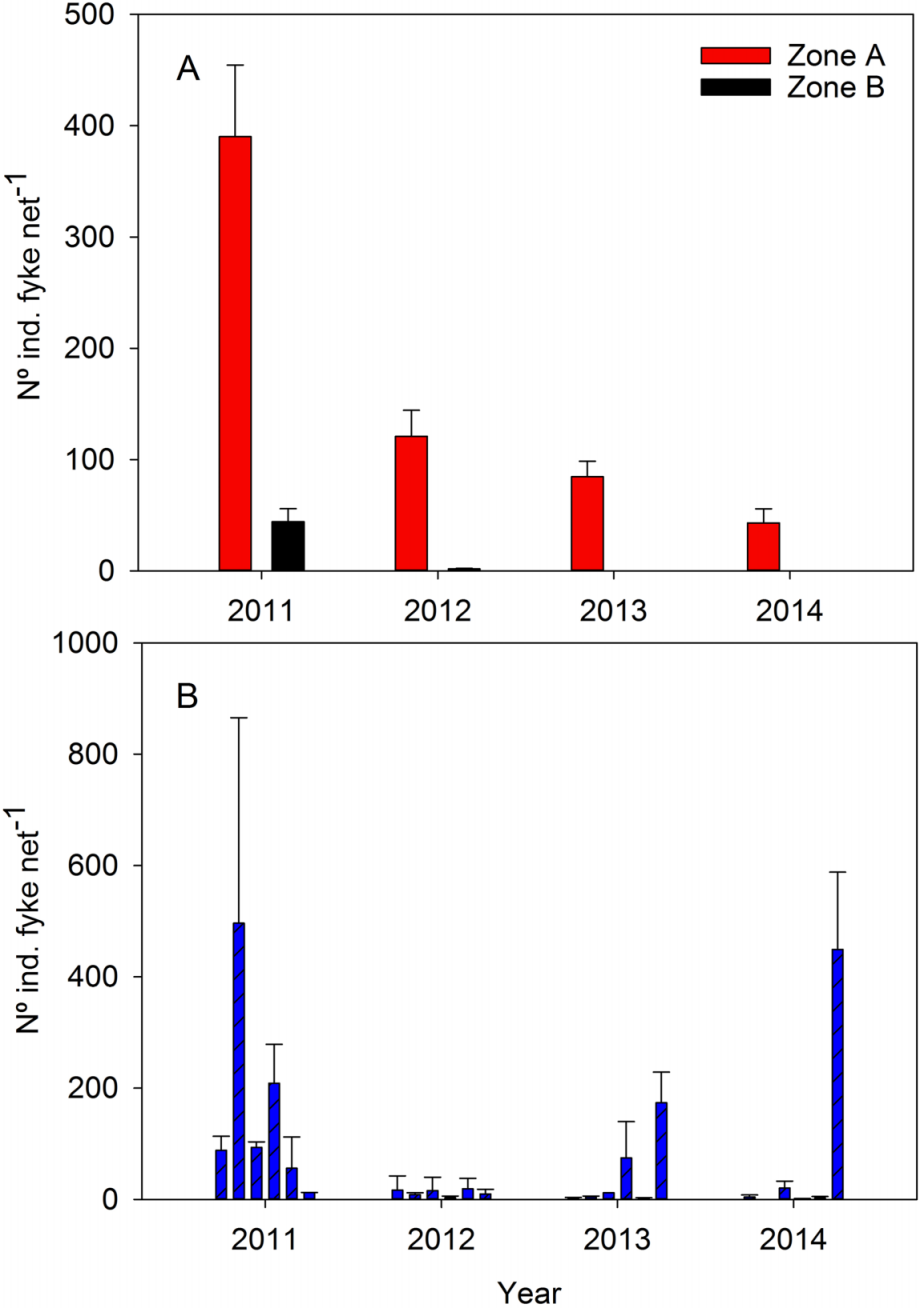
Number of *A. iberus* individuals captured within fyke nets. (A) The two study zones. (B) The six isolated ponds located within the zone A. Error bars are the standard error.

The abundance of *A. iberus* displayed significant differences among years (*F*_3, 439_ = 47.25, *P* < 0.001), with the highest numbers observed in Zone A in 2011, and no significant effects between years after restoration (in SNK: 2011 > 2012 = 2013 = 2014). There were also significant differences between zones (*F*_1, 439_ = 47.25, *P* < 0.001; in SNK: Zone A > Zone B), with sharper differences after 2011 (Year × Zone interaction; *F*_3, 439_ = 47.25, *P* = 0.001) (Fig. 5A). The number of individuals in reservoir ponds also showed a significant decline after 2011 and then increased abundances in 2013–2014 (5,460, 369, 1,511, and 1,436 individuals in total within the six reservoir ponds, respectively from 2011 to 2014; *F*_3, 48_ = 13.01, *P* < 0.001) (Fig. 5B). Significant effects were observed among ponds (*F*_5, 48_ = 4.6, *P* = 0.0015), and among ponds and time (Year × Pond interaction; *F*_15, 48_ = 6.85, *P* < 0.001).

Results of the information–theoretic framework analysis provided predictive models of the effects of measured environmental variables on the abundance of *A. iberus* in each study area. Considering all the data collected from 2012 to 2014, the AICc-based model selection suggested from 7–44 models for the whole area that could be considered as plausible models (*Δ*AICc < 7) to explain variability in *A. iberus* abundance. Consistently, the best AICc model was that containing distance to the sea and *A. boyeri*. Only in the 2014 model were the abundance of the crab *C. maenas* and salinity also found to be significant variables in the model (Table 4).

**Table 4:** Importance of environmental and biotic variables in ruling variations of *A. iberus* abundance at each year after restoration works (2012–2014), results from the information-theoretic analyses; see Table 2 for details.

Model parameter	2012			2013			2014		
	*N* = 84, *r* = 0.87			*N* = 81, *r* = 0.83			*N* = 172, *r* = 0.71		
	*β*	SP	Bias	*β*	SP	Bias	*β*	SP	Bias
Intercept	−0.739		16.210	−5.324		0.350	−15.889		−0.578
AB	**0.978**	**1.000**	**−0.093**	**1.103**	**1.000**	**0.080**	**0.744**	**1.000**	**0.058**
CM	−0.049	0.135	−9.311	−0.305	0.194	0.472	**−0.411**	**0.521**	**0.103**
Depth (cm)	0.927	0.196	−0.215	0.936	0.273	0.350	0.115	0.205	0.777
ODO (mg/L)	−4.987	0.353	−0.277	−1.048	0.226	1.511	−0.893	0.439	−0.273
Dist. to sea (m)	**1.890**	**1.000**	**0.293**	**2.545**	**1.000**	**−0.017**	**1.226**	**0.962**	**−0.016**
PM	−0.171	0.159	1.468	0.050	0.166	−0.147	−0.254	0.251	−0.072
Riparian veg. (%)	0.023	0.181	−0.029	−0.006	0.173	−9.191	−0.014	0.235	−0.104
**Salinity**	0.523	0.167	3.231	0.547	0.178	−0.874	**9.516**	**0.750**	**−0.143**
Submerged veg. (%)	−0.023	0.185	−1.499	0.011	0.168	0.876	0.001	0.203	10.589
Water T °C	−3.699	0.442	−0.590	−2.756	0.333	−0.576	3.609	0.400	−0.180

**Note:**

Parameters included in the best model are highlighted in bold.

## Discussion

Restoration efforts considerably enhanced the number of hydrological connections with the sea, providing additional submerged habitat (see aerial photographs in Supplemental Information 3) and improved esthetic landscape features within the salt marsh. Although there were few overall differences in fish diversity and richness among study years, a significantly higher number of species was able to reach semi-isolated areas after restoration. Areas adjacent to the sea showed a decrease in the abundance of ES whereas that of marine species increased, evidencing a relationship between habitat characteristics and fish community indicators (França et al., 2012). Yet the overall community structure showed little influence of restoration, possibly because of the low number of species present in the area and/or the fact that assemblages may require longer than 2–3 years to recover (Lorenz & Serafy, 2006; Warren et al., 2002). However, stronger effects may occur during other seasons not examined in this study. In fact, according to Smokorowski, Withers & Kelso (1998), only ca. 5% of published projects targeting increases in fish populations achieve enhanced fish production, even though ca. 98% of them reach habitat quality goals. The population of *A. iberus* was also strongly affected by distance from the sea, with higher numbers of individuals being found farther into the marsh, suggesting high site fidelity (Oliva-Paterna, Torralva & Fernández-Delgado, 2006). Abundances showed an important decrease after restoration which is mostly attributed to interannual differences in recruitment success (Fernández-Delgado et al., 1988; Vargas & De Sostoa, 1996), and to a lesser extent, to dispersal into new available habitat within the salt marsh and into the Alfacs bay. Overall, we are confident that enhancement of the hydrologic connectivity was the best strategy for habitat restoration in order to achieve a more natural functioning of the system while still hosting a large population of *A. iberus*.

### Effects of isolation degree in fish communities

The capacity for biological recovery following habitat rehabilitation has often been discussed as a function of isolation, with more isolated environments displaying slower rates of recovery for both invertebrate and fish communities (Fuchs & Statzner, 1990; Bond & Lake, 2003). For instance, increases in the abundance of trout (*Salmo trutta*) and brook trout (*Salvelinus fontinalis*) after habitat rehabilitation occurs through dispersal of individuals from adjacent areas and over a relatively large scale (Gowan & Fausch, 1996). However, recovery effects may also depend on habitat characteristics and on the way that target species exploit the habitat resources (Lorenz & Serafy, 2006). In our study, increasing distance from the sea was shown to be the most important variable explaining fish diversity and community structure before and after restoration of habitat connections. In 2011, fish diversity showed a negative relationship with distance, due to the overall dominance of ES (mostly *A. iberus* and *P. microps*), particularly in isolated areas, and a very low presence of marine species which were constricted to areas immediately adjacent to the sea. In contrast, a positive relationship with distance was observed in 2013–2014, apparently resulting from enhanced accessibility of individuals to remote areas of the saltmarsh, although with little differences in the relative abundances of ecological groups. Besides, the abundance of ES in areas adjacent to the sea decreased considerably after restoration, possibly as a response to increased predation risk (Mittelbach, 1986) due to higher numbers of marine species in the area (mostly juveniles of *S. aurata*). Similar observations were reported by Franco et al. (2006) who found quantitative dominance of resident ES within confined shallow environments, followed by marine species in more open habitats. In the particular case of 2012 (first year after restoration), the negative effect of distance on fish diversity might be attributed to a period of recovery after restoration works.

Among common species in the Ebro Delta, mullets, and European eel have a seasonal cycle marked by inshore migration to marshes and estuaries during the spring (Poole, Reynolds & Moriarty, 1990; Lebreton et al., 2011) and these taxa tended to be more abundant farther from the sea. Similarly, small species such as the common goby (*P. microps*) and the sand smelt (*A. boyeri*), which can be permanent residents and/or enter salt marshes in large numbers (Veiga et al., 2006; Green et al., 2009), were also more abundant at greater distances to the sea (Zone A). For the Spanish toothcarp, higher numbers were consistently observed farther into the marsh, possibly because of lower accessibility to predators compared to areas adjacent to the bay (Clavero, Blanco-Garrido & Prenda, 2007), or higher salinities (by ca. 10 units) also decreasing the presence of competitors and predators (Alcaraz & García-Berthou, 2007b; Alcaraz, Pou-Rovira & García-Berthou, 2008). For instance, the green crab was more abundant in salt marsh areas adjacent to the sea (up to 19 individuals per fyke net), and showed significant negative effects on *A. iberus* abundances in regression models (2011 and 2014). These negative relationships may be due to substantial digging and cutting of the submerged vegetation (Ropes, 1968; Davis, Short & Burdick, 1998), which can result in a decrease in the hatching success of *A. iberus*, which typically deposits egg masses on aquatic plants (Clavero, Blanco-Garrido & Prenda, 2007). Positive associations with *A. boyeri* abundances were observed throughout the study, although they might be indicative of similar habitat requirements (Clavero et al., 2005) rather than a causal relationship. The sea bream (*S. aurata*) was found in higher abundances closer to the sea, possibly due to more optimal growth, osmoregulation and metabolic efficiency at brackish and seawater salinities (Laiz-Carrión et al., 2005). The remaining taxa, including members of the Syngnathidae, as well as *S. pavo* and *Gobius geniporus* were mostly ES (Franco et al., 2006) but were present in low abundance and showed no relationship with distance to the sea. Overall, enhancing the hydrological connections favored the free movement of fish farther into the salt marsh, although overall differences in species richness and diversity appear to be primarily driven by interannual variability (see also Neill et al., 1994).

### Interannual variability in fish community

Year was also found to be an important factor driving fish community structure within the study salt marsh, possibly resulting from variability in the sign of some environmental variables (e.g., salinity and temperature) through time among other indeterminate causes. Populations trends for the three most abundant fish species (*A. iberus*, *P. microps*, and *A. boyeri*) and the green crab (*C. maenas*) suggest that periods longer than three years might be necessary to complete the recovery of estuarine assemblages (from five to 21 years according to Warren et al., 2002), although some negative effects of restoration works on the abundance of individuals might have also occurred. *A. iberus* was the most abundant species (0–3,508 individuals per fyke net), but numbers declined by ca. 81% from 2011 to 2012–2014, possibly due to dispersal to newly available salt marsh areas and/or the Alfacs Bay and to differences in interannual recruitment (see later), although some negative effects of restoration works cannot be excluded. For *P. microps*, a similar decrease was observed after restoration (ca. 92%), with similar patterns of decline also observed within reservoir ponds (from ca. 15.2 to 1.2 individuals per fyke net from 2011 to 2014, respectively for each year; see Supplemental Information 1) which suggests the effects of environmental factors (Dolbeth et al., 2007) and/or dispersal. For instance, salinity and oxygen levels were found to be significant factors during the study and are known to affect the use and selection of microhabitat patches by fish species, particularly during early stages of development (Baltz, Rakocinski & Fleeger, 1993; Alcaraz & García-Berthou, 2007b). Equally, the cover of riparian vegetation was also an important factor for the fish community, possibly because it enhances structural refuges, thus increasing species richness by harboring different small benthic species (Sabo et al., 2005). Yet as for *A. iberus*, potential negative effects of restoration works on this species, through enhanced turbidity or disturbance of the benthic habitat, cannot be discounted. In contrast, other common species such as *A. boyeri* and *C. maenas* showed similar overall abundances throughout the study (1–3 and 1–5 individuals per fyke net, respectively), which might be attributed to the use of distinctive trophic resources or other sources of natural variation (Pihl, 1985; Vizzini & Mazzola, 2005).

### Spanish toothcarp population

The population of *A. iberus* in the study area is one of the largest populations so far reported for the Spanish Mediterranean coast (Clavero, Blanco-Garrido & Prenda, 2006; Pou-Rovira et al., 2008; Rodríguez-Climent et al., 2012), and showed to be mostly determined by distance to the sea throughout the study period, which is coherent with the preference of the species for isolated areas (Oliva-Paterna, Torralva & Fernández-Delgado, 2006; Verdiell-Cubedo et al., 2013), independently of the presence of connections. Yet other factor commonly reported as decisive for determining the abundance of individuals such as the cover of submerged vegetation (e.g., Alcaraz, Pou-Rovira & García-Berthou, 2008), was not found to be significant, presumably due to the low spatial variability in the percent cover of submerged vegetation across the sampling area (from 1.7 to 7.2%).

The population reached the highest values in 2011 prior to restoration works. Although the turbidity associated to restoration efforts might have contributed to this pattern, abundances of individuals in isolated reservoir ponds also experienced a sharp decline after 2011, which suggest the influence of local natural factors such as climatic conditions and/or density-dependent population dynamics. Seasonal and interannual fluctuations of over >90% (Pou-Rovira et al., 2004) may be caused by temperature (low winter values, and/or differences in the duration of the reproductive window) and/or heavy rain and flooding events (stress due rapid changes in salinity and/or changes in prey availability), among other possibilities (Clavero, Blanco-Garrido & Prenda, 2007; Green et al., 2009). In particular in the study area, high-rainfall-driven episodic flood events during the winter period might have caused fish mortality (e.g., entry of competitors and predators) and/or favored the movement of some individuals to other adjacent areas outside the reservoir ponds. For instance, although only one individual of *C. maenas* and *A. anguilla* one were found in isolated ponds in 2011, increased numbers were detected in 2012 (seven individuals of each species) and 2013 (107 and 29, respectively for *C. maenas* and *A. anguilla*), and none in 2014, evidencing some flux of individuals during flood events. The large temporal fluctuations in the abundance of *A. iberus* were also consistent with the short life cycle of the species, which was found to be exclusively comprised by age 1^+^ individuals (Length based Cohort Analysis with the FiSAT II Software; see Supplemental Information 2), in agreement with previous age determinations in the Ebro Delta (García-Berthou & Moreno-Amich, 1992; Vargas & De Sostoa, 1996).

Among other aspects influencing the abundance of *A. iberus*, dilution of individuals due to increases in submerged area after restoration (11.3 and 27.1% in Zone A and B, respectively) is also possible, and would have required a greater sampling effort. In addition, some dispersion of individuals to other salt marsh areas adjacent to the restoration site might have occurred along the vegetation fringe with the Alfacs Bay. *A. iberus* populations from the Ebro Delta (Tancada, Canal Vell, and Gola del Migjorn) have been shown a genetic variability of 35.3% with respect to the original source and differences increase when compared to other geographical locations (Araguas et al., 2007). This variability provides indirect evidence of fish dispersion and suggests that, in the long term, enhanced salt marsh connectivity may help to promote the genetic diversity of the species. Yet given the small home range (usually 0–10 s of m) suggested for *A. iberus* and YOY of the Cyprinodontidae family (Alcaraz, Pou-Rovira & García-Berthou, 2008; Able, Hagan & Brown, 2006) and the general lack of records for this species in open marine waters (e.g., Oliva-Paterna, 2006), effective dispersion is possibly very low (i.e., not responsible for observed patterns of populations) and constricted to extreme events such as large storms.

## Conclusion

Restoration efforts created hydrological conditions and landscape configuration more similar to those occurring in the study habitat prior to human alteration in the 19th century, except for the traversing road (Supplemental Information 3). Enhanced fish diversity and abundance goals were not met, suggesting that the duration of the study period might have been insufficient for detecting the growth of populations (Lorenz & Serafy, 2006; Warren et al., 2002). Yet the combined application of community metrics (species richness and diversity) and ecological guild approach (review by Pérez-Domínguez et al., 2012) allowed the identification of more subtle changes in distribution of assemblages (i.e., enhanced number of species in remote areas and higher abundances of marine species in areas adjacent to the sea) that pointed to a gradual improvement in the ecological-quality status following restoration. For the two of the most abundant species, *A. iberus* and *P. microps*, restoration efforts were followed by a significant decrease in the abundance of individuals, but similar patterns were also detected in isolated reservoir ponds, thus suggesting the undergoing of natural factors (climatic factors, populations’ dynamics, etc.) rather than restoration works themselves. In particular, episodic flooding of isolated ponds during winter storms cannot be discarded, and might have been a process involved in fish mortality and/or allowing the movement of some individuals toward other adjacent areas of the salt marsh, at least during episodes of extreme climatic conditions. In the long term, given the marked preference of *A. iberus* for isolated environments, enhancement of hydrological connections may benefit the occasional dispersion of individuals and favor the genetic diversity of the species.

## Supplemental Information

10.7717/peerj.3009/supp-1 Supplemental Information 1 Fish abundances per year and zone. Abundances of fish species at each study year and zone. Each row indicates numbers of individuals within a given fykenet.

10.7717/peerj.3009/supp-2 Supplemental Information 2 Supplemental data in cohort analysis of *Aphanius iberus*.

10.7717/peerj.3009/supp-3 Supplemental Information 3 Annex I. Temporal changes in the study area. (A) In 1860, the northern coast of the Alfacs Bay (see also Fig. 1) was fully fingered by salt marsh which connected with a much larger lacunar system (nowadays the Encanyissada and Tancada lagoons constitute remaining portions). (B) In 1927 to (C) 1954, the salt pans were already built but the loss of salt marsh area increases progressively. (D) Full view of the abandoned fish farm before connectivity enhancement in 2011, and (E) current aspect of the study area after rehabilitation works.

## Additional Information and Declarations

### Field Study Permissions

The following information was supplied relating to field study approvals (i.e., approving body and any reference numbers): A field permit for fish capture was granted by the Ebro Delta Natural Park, which also provided logistical support and supervision at each sampling event once per year during four consecutive years (2011–2014). No individuals were sacrificed for the purpose of this study, and all fish were released at the site of capture immediately after species identification.

### Data Deposition

The following information was supplied regarding data availability: The raw data has been supplied as Supplementary Dataset Files.

## References

[ref-1] Able KW, Hagan SM (2000). Effects of common reed (*Phragmites australis*) invasion on marsh surface macrofauna: response of fishes and decapod crustaceans. Estuaries.

[ref-2] Able KW, Hagan SM, Brown SA (2006). Habitat use, movement, and growth of young-of-the-year *Fundulus* spp. in southern New Jersey salt marshes: comparisons based on tag/recapture. Journal of Experimental Marine Biology and Ecology.

[ref-3] Alcaraz C, Bisazza A, García-Berthou E (2008). Salinity mediates the competitive interactions between invasive mosquitofish and an endangered fish. Oecologia.

[ref-4] Alcaraz C, García-Berthou E (2007a). Life history variation of invasive mosquitofish along a salinity gradient. Biological Conservation.

[ref-5] Alcaraz C, García-Berthou E (2007b). Food of an endangered cyprinodont (*Aphanius iberus*): ontogenetic diet shift and prey electivity. Environmental Biology of Fishes.

[ref-6] Alcaraz C, Pou-Rovira E, García-Berthou E (2008). Use of a flooded salt marsh habitat by an endangered cyprinodontid fish (*Aphanius iberus*). Hydrobiologia.

[ref-7] Araguas RM, Roldán MI, García-Marín JL, Pla C (2007). Management of gene diversity in the endemic killifish *Aphanius iberus*: revising Operational Conservation Units. Ecology of Freshwater Fish.

[ref-8] Baltz DM, Rakocinski C, Fleeger JW (1993). Microhabitat use by marsh-edge fishes in a Louisiana estuary. Environmental Biology of Fishes.

[ref-9] Beck MW, Heck KL, Able KW, Childers D, Childers D, Eggleston D, Gillanders B, Halpern BS, Hayes CG, Hoshino K, Minello TJ, Orth RO, Sheridan PF, Weinstein MP (2003). The role of near shore ecosystems as fish and shellfish nurseries. Issues in Ecology.

[ref-10] Benito X, Trobajo R, Ibáñez C (2014). Modelling habitat distribution of Mediterranean coastal wetlands: the Ebro Delta as case study. Wetlands.

[ref-11] Bond NR, Lake PS (2003). Local habitat restoration in streams: constraints on the effectiveness of restoration for stream biota. Ecological Management and Restoration.

[ref-12] Burnham KP, Anderson DR (2002). Model Selection and Multimodel Inference: A Practical Information-Theoretic Approach.

[ref-13] Ceballos G, Rodríguez P, Medellín RA (1998). Assessing conservation priorities in megadiverse Mexico: mammalian diversity, endemicity, and endangerment. Ecological Applications.

[ref-14] Clarke KR, Gorley GR (2006). PRIMER v6: User Manual/Tutorial.

[ref-15] Clavero M, Blanco-Garrido F, Prenda J (2006). Monitoring small fish populations in streams: A comparison of four passive methods. Fisheries Research.

[ref-16] Clavero M, Blanco-Garrido F, Prenda J (2007). Population and microhabitat effects of interspecific interactions on the endangered Andalusian toothcarp (*Aphanius baeticus*). Environmental Biology of Fishes.

[ref-17] Clavero M, Blanco-Garrido F, Zamora L, Prenda J (2005). Size-related and diel variations in microhabitat use of three endangered small fishes in a Mediterranean coastal stream. Journal of Fish Biology.

[ref-18] Davis RC, Short FT, Burdick DM (1998). Quantifying the effects of green crab damage to eelgrass transplants. Restoration Ecology.

[ref-19] Doadrio I (2002). Atlas y Libro Rojo de los Peces Continentales de España.

[ref-20] Dolbeth M, Martinho F, Leitão R, Cabral H, Pardal MA (2007). Strategies of *Pomatoschistus minutus* and *Pomatoschistus microps* to cope with environmental instability. Estuarine Coastal and Shelf Science.

[ref-21] Fernández-Delgado C, Hernando JA, Herrera M, Bellido M (1988). Age, growth and reproduction of *Aphanius iberus* (Cuv. & Val., 1846) in the lower reaches of the Guadalquivir river (south-west Spain). Freshwater Biology.

[ref-22] França S, Vasconcelos RP, Reis-Santos P, Fonseca VF, Costa MJ, Cabral HN (2012). Vulnerability of Portuguese estuarine habitats to human impacts and relationship with structural and functional properties of the fish community. Ecological Indicators.

[ref-23] Franco A, Franzoi P, Malavasi S, Riccato F, Torricelli P, Mainardi D (2006). Use of shallow water habitats by fish assemblages in a Mediterranean coastal lagoon. Estuarine, Coastal and Shelf Science.

[ref-24] Fuchs U, Statzner B (1990). Time scales for the recovery potential of river communities after restoration: lessons to be learned from smaller streams. Regulated Rivers: Research and Management.

[ref-25] García-Berthou E, Moreno-Amich R (1992). Age and growth of an Iberian cyprinodont, *Aphanius Iberus* (Cuv. & Val.), in its most northerly population. Journal of Fish Biology.

[ref-26] Gedan KB, Silliman BR, Bertness MD (2009). Centuries of human-driven change in salt marsh ecosystems. Annual Review of Marine Science.

[ref-27] Gowan C, Fausch KD (1996). Long-term demographic responses of trout populations to habitat manipulation in six Colorado streams. Ecological Applications.

[ref-28] Green BC, Smith DJ, Earley SE, Hepburn LJ, Underwood GJC (2009). Seasonal changes in community composition and trophic structure of fish populations of five salt marshes along the Essex coastline, United Kingdom. Estuarine Coastal and Shelf Science.

[ref-29] Halpern BS, Walbridge S, Selkoe KA, Kappel CV, Micheli F, D’Agrosa C, Bruno JF, Casey KS, Ebert C, Fox HE, Fujita R, Heinemann D, Lenihan HS, Madin EMP, Perry MT, Selig ER, Spalding M, Steneck R, Watson R (2008). A global map of human impact on marine ecosystems. Science.

[ref-30] Ibáñez C, Alcaraz C, Caiola N, Rovira A, Trobajo R, Alonso M, Duran C, Jiménez PJ, Munné A, Prat N (2012). Regime shift from phytoplankton to macrophyte dominance in a large river: top-down versus bottom-up effects. Science of the Total Environment.

[ref-31] Ibáñez C, Canicio A, Day JW, Curcó A (1997). Morphologic evolution, relative sea-level rise and sustainable management of water and sediment in the Ebre Delta. Journal of Coastal Conservation.

[ref-32] Koch EW, Barbier EB, Silliman BR, Reed DJ, Perillo GME, Hacker SD, Granek EF, Primavera JH, Muthiga N, Polasky S, Halpern BS, Kennedy CJ, Kappel CV, Wolanski E (2009). Non-linearity in ecosystem services: temporal and spatial variability in coastal protection. Frontiers in Ecology and the Environment.

[ref-33] Koroleff F, Grasshoff K, Kremling K, Erhardt M, Osterroth C (1977). Simultaneous persulfate oxidation of phosphorus and nitrogen compounds in water. Report of the Baltic Intercalibration Workshop.

[ref-34] Laiz-Carrión R, Sangiao-Alvarellos S, Guzmán JM, Martín del Río MP, Soengas JL, Mancera JM (2005). Growth performance of gilthead sea bream *Sparus aurata* in different osmotic conditions: implications for osmoregulation and energy metabolism. Aquaculture.

[ref-35] Lebreton B, Richard P, Parlier EP, Guillou G, Blanchard GF (2011). Trophic ecology of mullets during their spring migration in a European saltmarsh: a stable isotope study. Estuarine, Coastal and Shelf Science.

[ref-36] Lorenz JJ, Serafy JE (2006). Subtropical wetland fish assemblages and changing salinity regimes: implications for everglades restoration. Hydrobiologia.

[ref-37] Matthews GA, Minello TJ (1994). Technology and Success in Restoration Creation, and Enhancement of Spartina alterniflora Marshes in the United States.

[ref-38] Meynecke JO, Lee SY, Duke NC, Warnken J (2007). Relationships between estuarine habitats and coastal fisheries in Queensland, Australia. Bulletin of Marine Science.

[ref-39] Mittelbach G, Simenstad CA, Cailliet GM (1986). Predator-mediated habitat use: some consequences for species interactions. Contemporary Studies on Fish Feeding: The Proceedings of GUTSHOP’84.

[ref-40] Neill WH, Miller JM, Van Der Veer HW, Winemiller KO (1994). Ecophysiology of marine fish recruitment: a conceptual framework for understanding interannual variability. Netherlands Journal of Sea Research.

[ref-41] Noss RF (2000). High-risk ecosystems as foci for conserving biodiversity and ecological integrity in ecological risk assessments. Environmental Science and Policy.

[ref-42] Oliva-Paterna FJ (2006). Biología y Conservación de *Aphanius iberus* (Valenciennes, 1846) en la Región de Murcia.

[ref-43] Oliva-Paterna FJ, Torralva M, Fernández-Delgado C (2006). Threatened fishes of the world: *Aphanius iberus* (Cuvier Valenciennes, 1846) (Cyprinodontidae). Environmental Biology of Fishes.

[ref-44] Pérez-Domínguez R, Maci S, Courrat A, Lepage M, Borja A, Uriarte A, Neto JM, Cabral H, Raykov VS, Franco A, Alvarez MC, Elliott M (2012). Current developments on fish-based indices to assess ecological-quality status of estuaries and lagoons. Ecological Indicators.

[ref-45] Pihl L (1985). Food selection and consumption of mobile epibenthic fauna in shallow marine areas. Marine Ecology Progress Series.

[ref-46] Poole WR, Reynolds JD, Moriarty C (1990). Observations on the silver eel migrations of the Burrishoole River system, Ireland, 1959–1988. Internationale Revue der gesamten Hydrobiologie und Hydrographie.

[ref-47] Portnoy JW, Giblin AE (1997). Biogeochemical effects of seawater restoration to diked salt marshes. Ecological Applications.

[ref-48] Pou-Rovira Q, Alcaraz C, Feo C, Zamora L, Vila-Gispert A, Carol Q, García-Berthou E, Moreno-Amich R (2004). Els peixos. Papers del Montgrí.

[ref-49] Pou-Rovira Q, Feo C, Valdivieso A, Canet F, Ferrer D (2008). Seguiment de la población de fartet (Aphanius Iberus) de les llacunes de la Pletera.

[ref-50] Pringle C (2003). What is hydrologic connectivity and why is it ecologically important?. Hydrological Processes.

[ref-51] Rodríguez-Climent S, Alcaraz C, Caiola N, Ibáñez C, Nebra A, Muñoz-Camarillo G, de Sostoa A (2012). Gillnet selectivity in the Ebro Delta coastal lagoons and its implication for the fishery management of the sand smelt, *Atherina boyeri* (Actinopterygii: Atherinidae). Estuarine Coastal and Shelf Science.

[ref-52] Ropes JW (1968). The feeding habits of the green crab, *Carcinus maenas* (L.). Fishery Bulletin.

[ref-53] Sabo JL, Sponseller R, Dixon M, Gade K, Harms T, Heffernan J, Jani A, Katz G, Soykan C, Watts J, Welter J (2005). Riparian zones increase regional species richness by harboring different, not more, species. Ecology.

[ref-54] Sheaves M (2009). Consequences of ecological connectivity: the coastal ecosystem mosaic. Marine Ecology Progress Series.

[ref-55] Smokorowski KE, Withers KJ, Kelso JRM (1998). Does habitat creation contribute to management goals? An evaluation of literature documenting freshwater habitat rehabilitation or enhancement projects. Canadian Technical Report of Fisheries and Aquatic Sciences.

[ref-56] Vargas MJ, De Sostoa A (1996). Life-history pattern of the Iberian toothcarp *Aphanius iberus* (Pisces, Cyprinodontidae) from a Mediterranean estuary, the Ebro delta (Spain). Netherlands Journal of Zoology.

[ref-57] Veiga P, Vieira L, Bexiga C, Sá R, Erzini K (2006). Structure and temporal variations of fish assemblages of the Castro Marim salt marsh, southern Portugal. Estuarine Coastal and Shelf Science.

[ref-58] Verdiell-Cubedo D, Ruiz-Navarro A, Torralva M, Moreno-Valcárcel R, Oliva-Paterna FJ (2013). Habitat use of an endangered cyprinodontid fish in a saline wetland of the Iberian Peninsula (SW Mediterranean Sea). Mediterranean Marine Science.

[ref-59] Vizzini S, Mazzola A (2005). Feeding ecology of the sand smelt *Atherina boyeri* (Risso 1810) (Osteichthyes, Atherinidae) in the western Mediterranean: evidence for spatial variability based on stable carbon and nitrogen isotopes. Environmental Biology of Fishes.

[ref-60] Warren RS, Fell PE, Rozsa R, Brawley AH, Orsted AC, Olson ET, Swamy V, Niering WA (2002). Salt marsh restoration in Connecticut: 20 years of science and management. Restoration Ecology.

[ref-61] Whittingham MJ, Swetnam RD, Wilson JD, Chamberlain DE, Freckleton RP (2005). Habitat selection by yellowhammers *Emberiza citrinella* on lowland farmland at two spatial scales: implications for conservation management. Journal of Applied Ecology.

[ref-62] Williams GD, Zedler JB (1999). Fish assemblage composition in constructed and natural tidal marshes of San Diego Bay: relative influence of channel morphology and restoration history. Estuaries.

[ref-63] Worm B, Barbier EB, Beaumont N, Duffy JE, Folke C, Halpern BS, Jackson JBC, Lotze HK, Micheli F, Palumbi SR, Sala E, Selkoe KA, Stachowicz JJ, Watson R (2006). Impacts of biodiversity loss on ocean ecosystem services. Science.

