# Supplementary material for: Effects of enhanced hydrological connectivity on Mediterranean salt marsh fish assemblages with emphasis on the endangered Spanish toothcarp (Aphanius iberus)
Source: PeerJ. 2017 Feb 28;5:e3009. doi: 10.7717/peerj.3009 (PMC5333551; doi:10.7717/peerj.3009)
Supplement: Supplemental Information 2 [file peerj-05-3009-s002.docx]

**Cohort Analysis**

Significantly different age classes (i.e., cohorts), were identified using the method of Bhattacharya (1967) available in the Fisat II software (FAO-ICCLARM Stock Assessment Tool). Then, a modal class progression analyses was applied in order to obtain an index that provides meaningful separation for values greater than 2 (Gayanilo et al. 1988).

The smallest recorded individual of *A. iberus* was 9 mm long and 77 mm for the largest, with ca. 86 % of the population being 22 to 36 mm long, consistently at each study year (i.e., 2011 to 2014, see Suppl. Fig. 1). Investigation of the number of *A. iberus* cohorts using the method of Bhattacharya (1967) showed that individuals at each of the three study years belonged to a single age group (i.e., separation indexes < 2; Suppl. Fig. 1).

**REFERENCES**

**Bhattacharya C. 1967.** A simple method of resolution of a distribution into Gaussian components. *Biometrics* **23:** 115-135.

**Gayanilo GC Jr, Sparre P, Pauly D. 2005.** FAPICLARM stock assessment tools II (FISAT II). Revised version. In: FAO, editor. *User’s guide*. FAO Computerized Information Series (Fisheries) No 8.Rome, Italy.

2011

2012

Frequency

2013

2014

Lt (mm)

| **Supp. Figure 1 Proportion of individuals of each size (Lt; total length) and number of cohorts (dotted line) resulting in modal class progression analyses within the entire study area (Zones A and B) from 2011 to 2014..** |
| --- |
