# Supplementary material for: Effects of enhanced hydrological connectivity on Mediterranean salt marsh fish assemblages with emphasis on the endangered Spanish toothcarp (Aphanius iberus)
Source: PeerJ. 2017 Feb 28;5:e3009. doi: 10.7717/peerj.3009 (PMC5333551; doi:10.7717/peerj.3009)
Supplement: Supplemental Information 3 — Temporal changes in the study area. (A) In 1860, the northern coast of the Alfacs Bay (see also Fig. 1) was fully fingered by salt marsh which connected with a much larger lacunar system (nowadays the Encanyissada and Tancada lagoons constitute remaining portions). (B) In 1927 to (C) 1954, the salt pans were already built but the loss of salt marsh area increases progressively. (D) Full view of the abandoned fish farm before connectivity enhancement in 2011, and (E) current aspect of the study area after rehabilitation works. [file peerj-05-3009-s003.pdf]

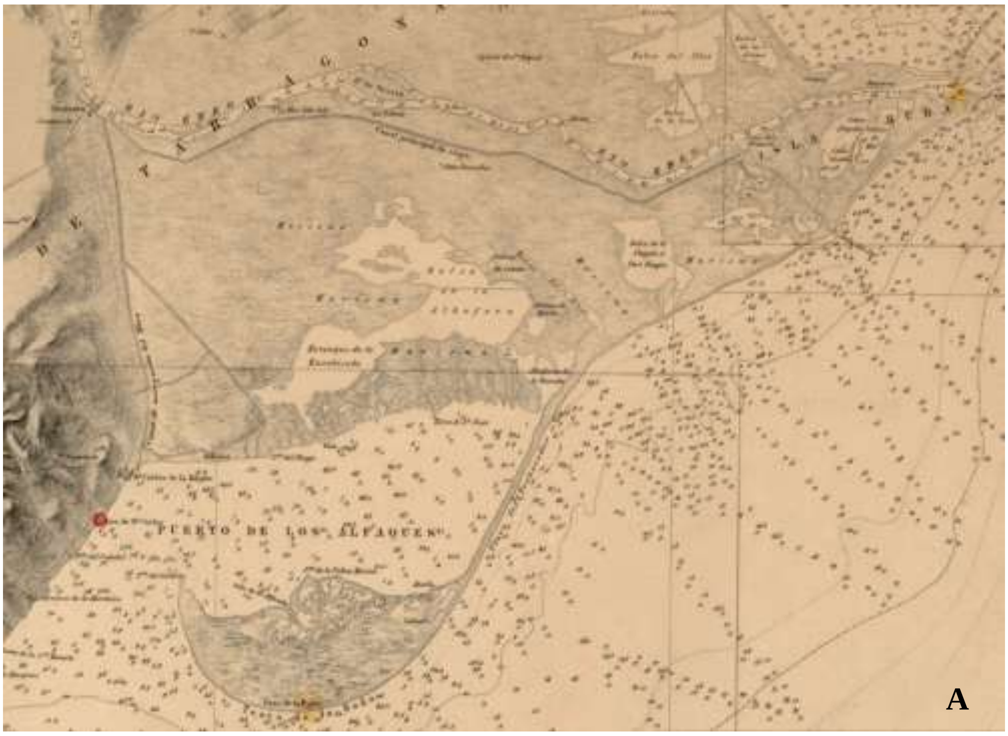
PUERTO DE LOS ALFAQUES
A

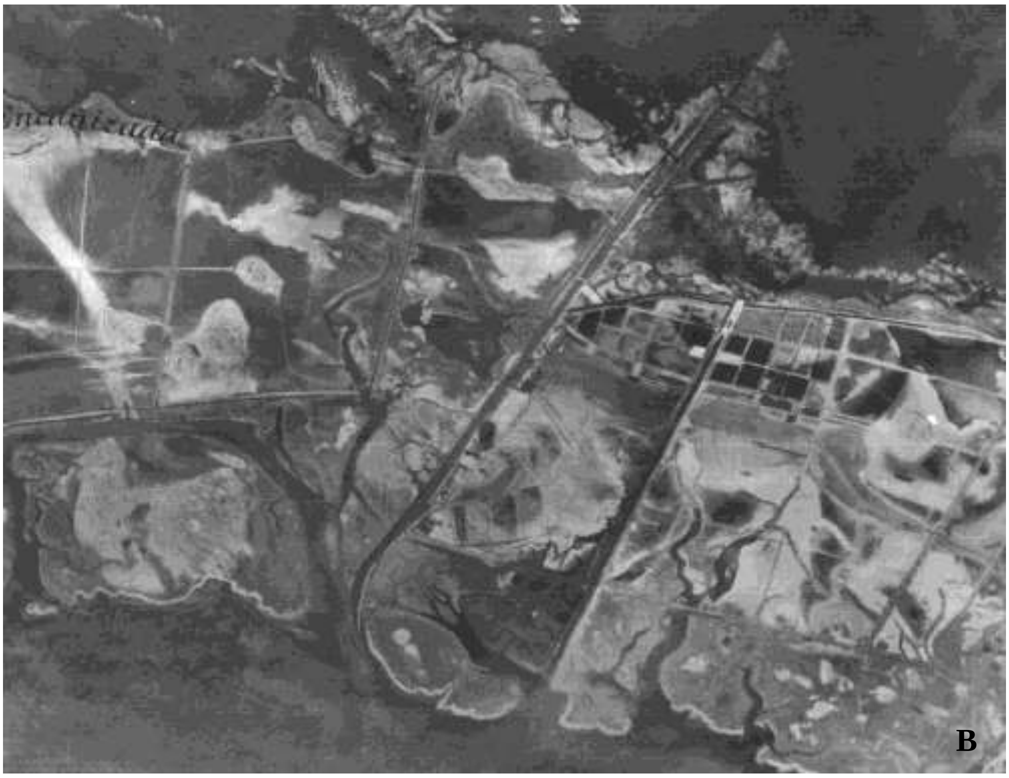
B

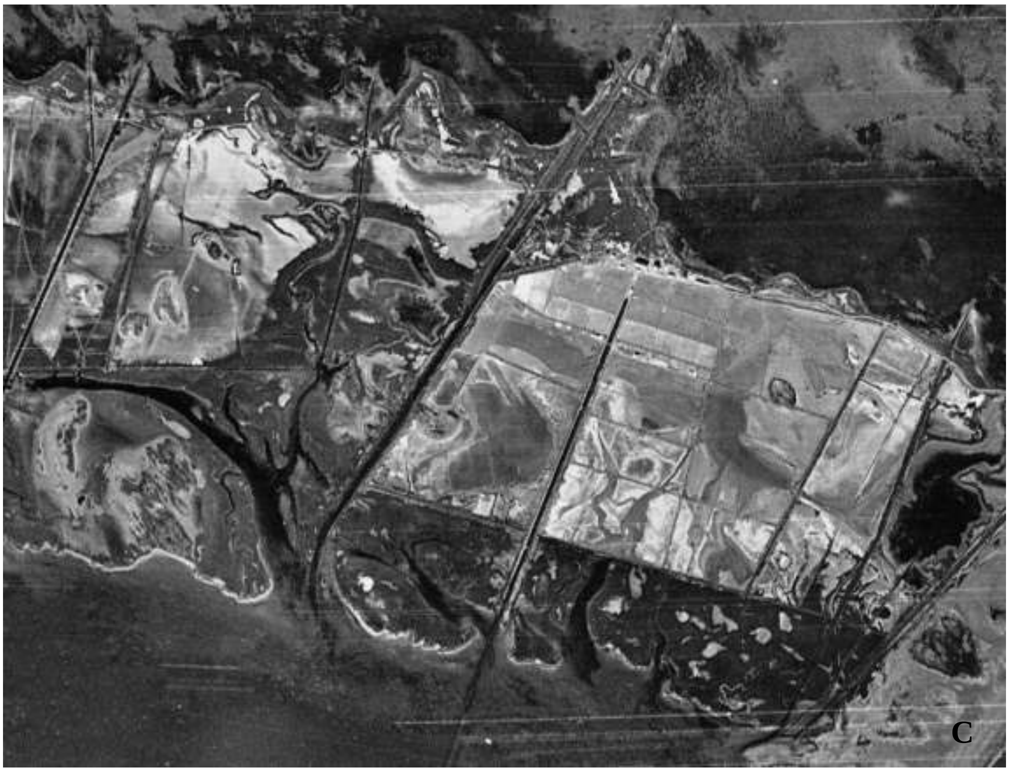
C

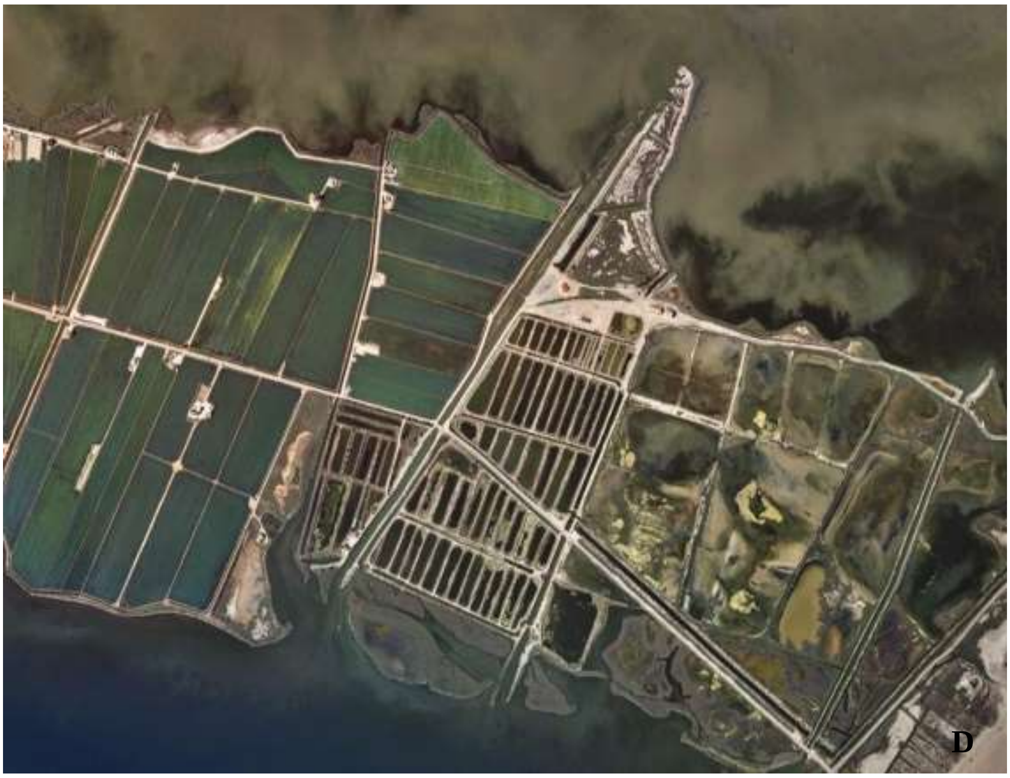
D

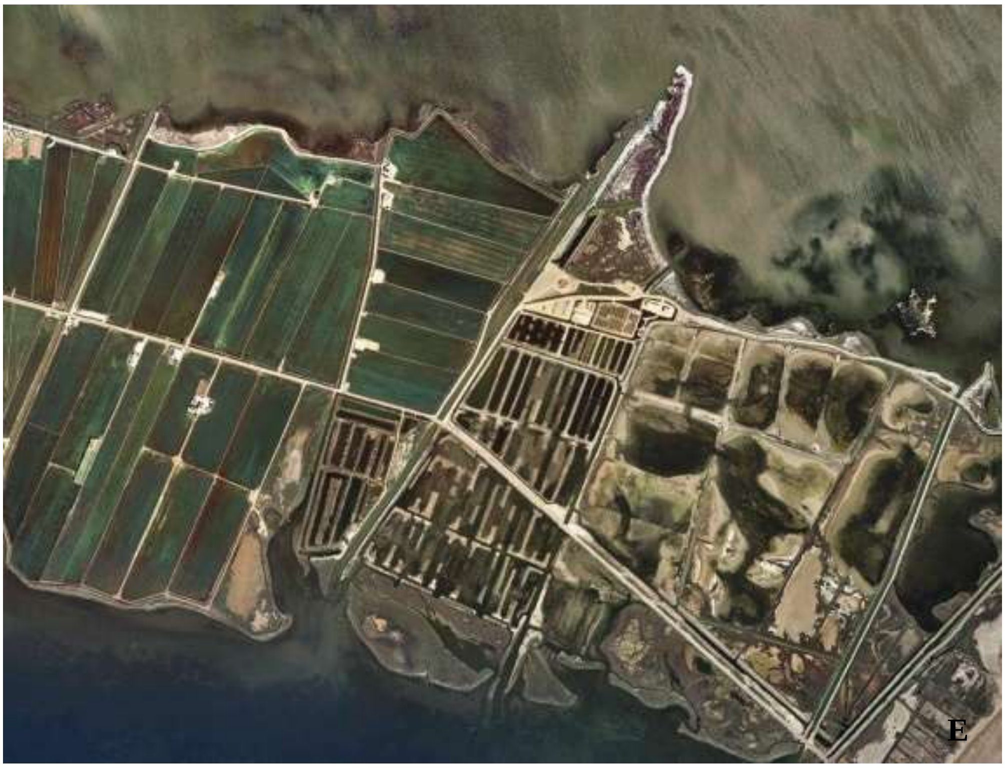

Annex I Temporal changes in the study area. A) In 1860, the northern coast of the Alfacs Bay (see also Fig. 1) was fully fingered by salt marsh which connected with a much larger lacunar system (nowadays the Encanyissada and Tancada lagoons constitute remaining portions). B) In 1927 to C) 1954, the salt pans were already built but the loss of salt marsh area increases progressively. D) Full view of the abandoned fish farm before connectivity enhancement in 2011, and E) current aspect of the study area after rehabilitation works.
